# Supplementary material for: Microbial Shift in the Enteric Bacteriome of Coral Reef Fish Following Climate-Driven Regime Shifts
Source: Microorganisms. 2021 Aug 11;9(8):1711. doi: 10.3390/microorganisms9081711 (PMC8398123; doi:10.3390/microorganisms9081711)
Supplement: Supplementary file 1 [file microorganisms-09-01711-s001.zip › Supplementary_files/Supplementary_material.pdf]

Supplementary Material

Microbial shift in the enteric bacteriome of coral reef fish following climate-driven regime shifts.

Marie-Charlotte Cheutin[1]*, Sébastien Villéger[1], Christina C. Hicks[2], James P. W. Robinson[2], Nicholas A. J. Graham[2], Clémence Marconnet[1], Claudia Ximena Ortiz Restrepo[1], Yvan Bettarel[1], Thierry Bouvier[1], Jean-Christophe Auguet[1]

[1] UMR MARBEC, Université de Montpellier, CNRS, Ifremer, IRD, Montpellier, France;
[2] Lancaster Environment Centre, Lancaster University, Lancaster LA1 4YQ, UK
* Correspondence: mccheutin@gmail.com

Supplementary Material

1 Supplementary Tables

Table S1 : Inventory of the species collected in recovered coral reefs (RCR) and macroalgae shifted reefs (MSR) with their corresponding diet and taxonomy.

Family	Species	Diet	RCR	MSR
Monacanthidae (n = 2)	*Cantherhines pardalis*	Omnivore	2	0
Carangidae (n = 2)	*Carangoides fulvoguttatus*	Invertivore	0	1
	Carangoides plagiotaenia	Invertivore	0	1
Serranidae (n = 3)	*Epinephelus spilotoceps*	Invertivore	1	0
	Aethaloperca rogaa	Piscivore	0	1
	Cephalopholis argus	Piscivore	1	0
Labridae (n = 8)	*Oxycheilinus digramma*	Invertivore	1	0
	Cheilinus fasciatus	Invertivore	1	0
	Cheilinus trilobatus	Invertivore	1	2
	Coris formosa	Invertivore	1	0
	Epibulus insidiator	Invertivore	1	0
	Hemigymnus fasciatus	Invertivore	1	0
Lethrinidae (n = 26)	*Lethrinus enigmaticus*	Invertivore	3	3
	Lethrinus mahsena	Invertivore	3	7
	Lethrinus microdon	Invertivore	1	4
	Lethrinus nebulosus	Invertivore	1	1
	Lethrinus variegatus	Invertivore	2	0
	Lethrinus xanthochilus	Invertivore	1	0
Lutjanidae (n = 14)	*Aprion virescens*	Piscivore	3	4
	Lutjanus fulviflamma	Invertivore	2	0
	Lutjanus gibbus	Invertivore	0	1
	Lutjanus kasmira	Invertivore	0	3
	Lutjanus vitta	Invertivore	1	0
Scarinae (n = 22)	*Calotomus carolinus*	Browsers	5	0
	Chlorurus sordidus	Scrapers	1	0
	Scarus falcipinnis	Scrapers	3	0
	Scarus ghobban	Scrapers	4	3
	Scarus niger	Scrapers	2	0
	Scarus psittacus	Scrapers	1	0
	Scarus rubroviolaceus	Scrapers	1	0
	Scarus tricolor	Scrapers	2	0
Siganidae (n = 17)	*Siganus argenteus*	Browsers	0	3
	Siganus sp.	Browsers	1	2
	Siganus sutor	Browsers	0	11
Acanthuridae (n = 5)	*Ctenochaetus binotatus*	Grazers	0	1
	Zebrasoma desjardinii	Grazers	0	4

Supplementary Material

Family	Species	Diet	RCR	MSR
Macroalgae (n = 29)	*Sargassum*	.	3	6
	Turf	.	11	9
Total			**61**	**67**

Supplementary Material

2 Supplementary Figures

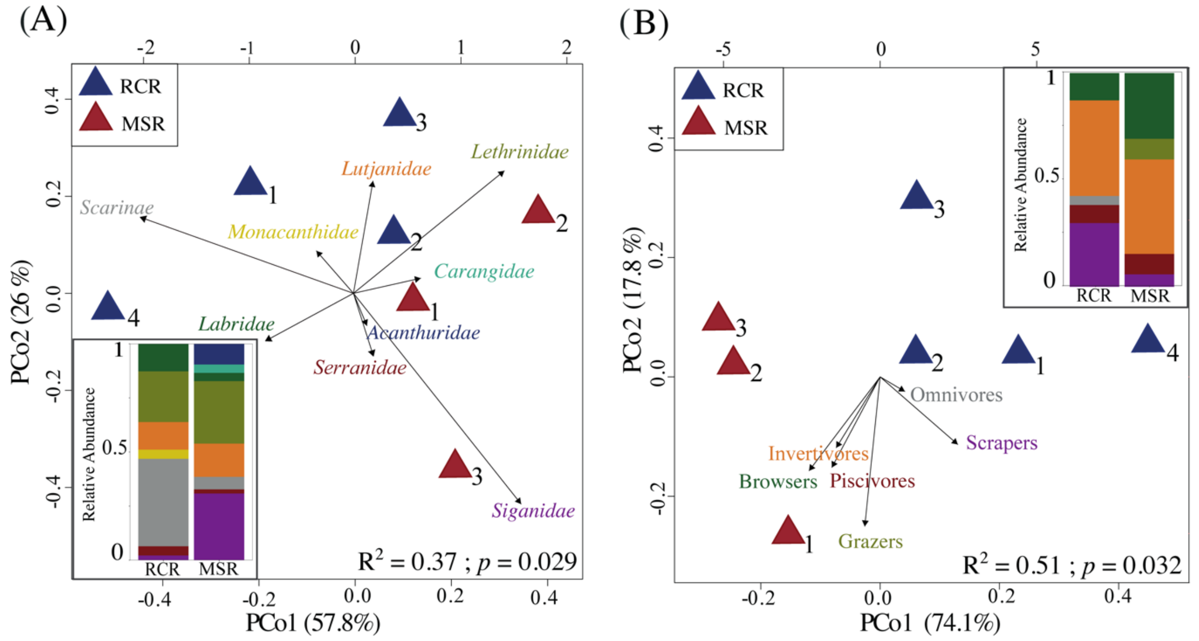

Figure S1: Principal coordinates analysis (PCoA) illustrating similarity of fish catch composition (**A**) and diets (**B**) between coral dominated reefs (RCR) and macroalgae shifted reefs (MSR).

Supplementary Material

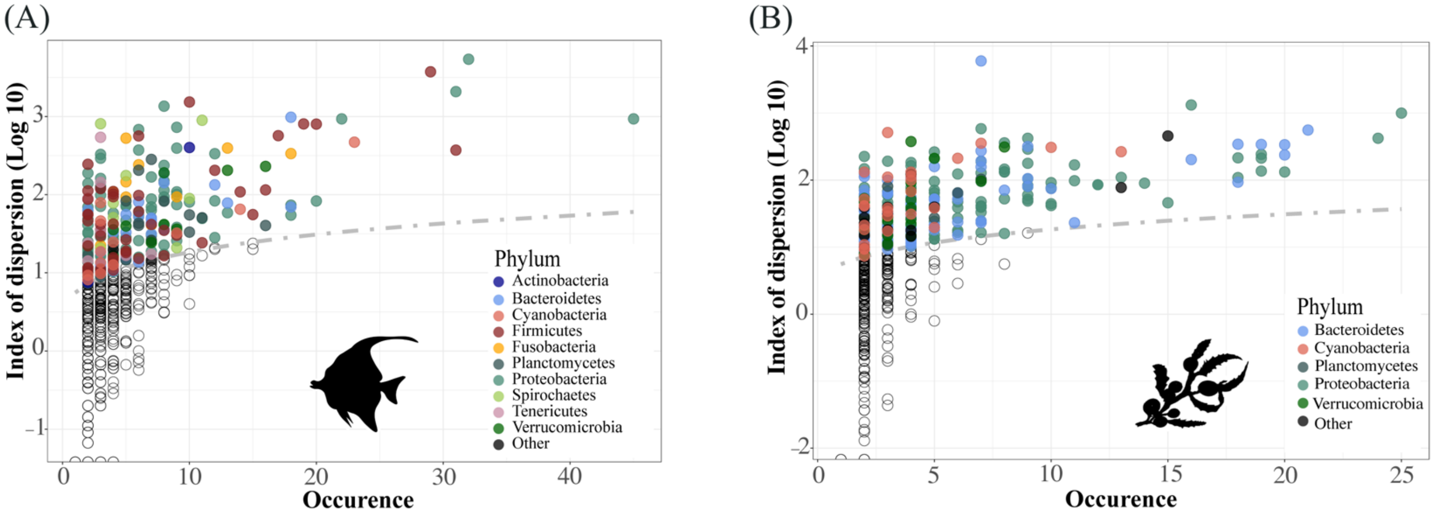

Figure S2: Species abundance distribution (SAD) pattern of bacterial ASVs in the fish gut (**A**) and macroalgae bacteriomes (**B**). Occurrence of each ASV is plotted against its dispersion index. The line depicts the 2.5% confidence limit of the Chi2 distribution: ASVs falling below this line follow a Poisson distribution and are randomly dispersed within bacteriomes.

Supplementary Material

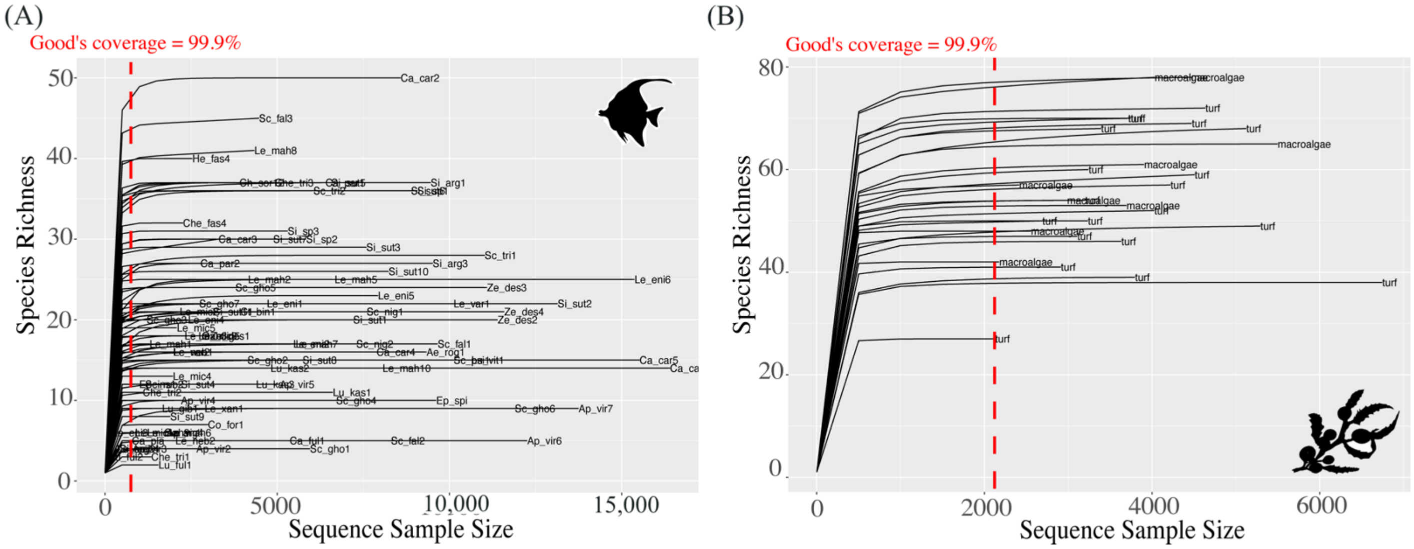

Figure S3: Rarefaction curves for each fish gut bacteriome (**A**) and macroalgae bacteriome (**B**) after a rarefaction at 1040 and 2621 sequences in each sample, respectively . The dotted line represents the number of sequences at which 99,9 % of the diversity is reached in all samples determined by the Good's coverage estimator.

Supplementary Material

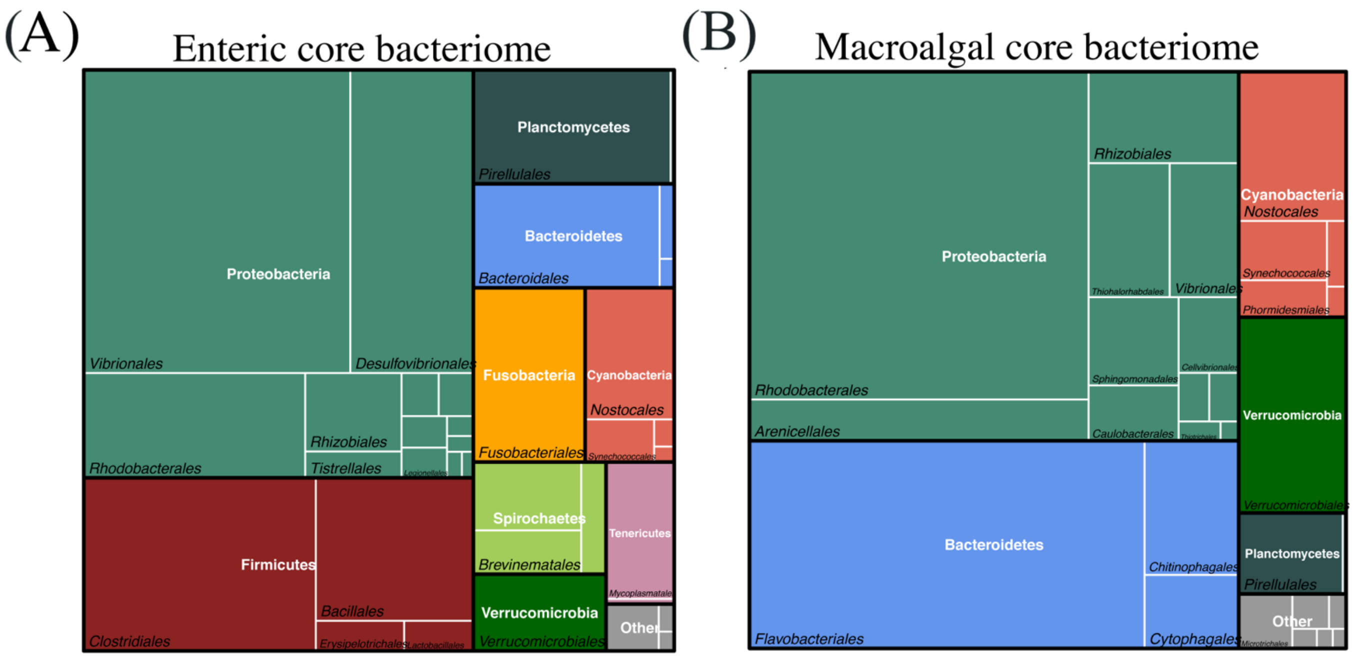

Figure S4: Treemaps of the constitutive phyla and their representative families in the enteric core bacteriome of reef fishes (**A**) and macroalgal core bacteriome (**B**).

Supplementary Material

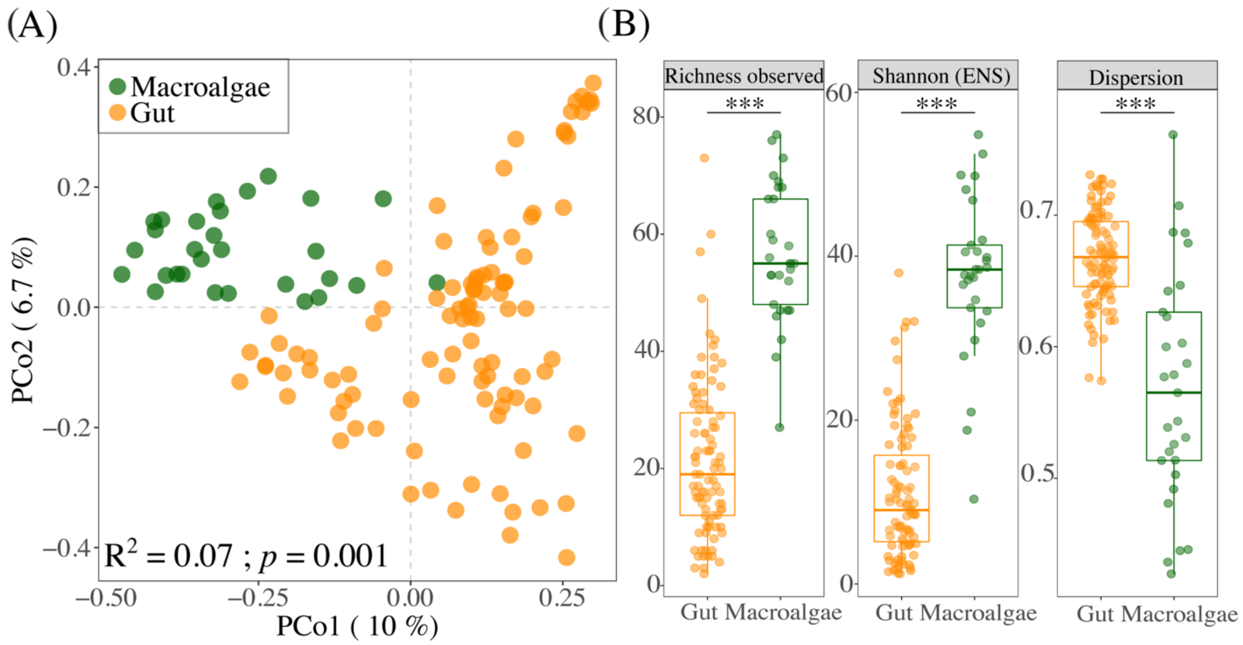

Figure S5: Beta and alpha diversity of fish gut and macroalgae bacteriomes. **(A)** Principal coordinates analysis (PCoA) plot illustrating Bray-Curtis distances between pairs of bacteriome samples. Bacteriomes are colored according to their origin: fish gut in orange and macroalgae in green. The p and R2 values of a PERMANOVA testing whether the origin of the samples have an influence are reported. **(B)** Alpha diversity is expressed as the observed richness and the Shannon's index H – exponentially transformed in effective number of species (ENS). The dispersion of the beta diversity corresponding to the distance to the centroïd for each sample type grouping is also shown. Horizontal brackets indicate pairs which differ significantly: *** ⩽ 0.001 ; ** ⩽ 0.01 ; * ⩽ 0.05) or not (NS.) with a Wilcoxon test.

Supplementary Material

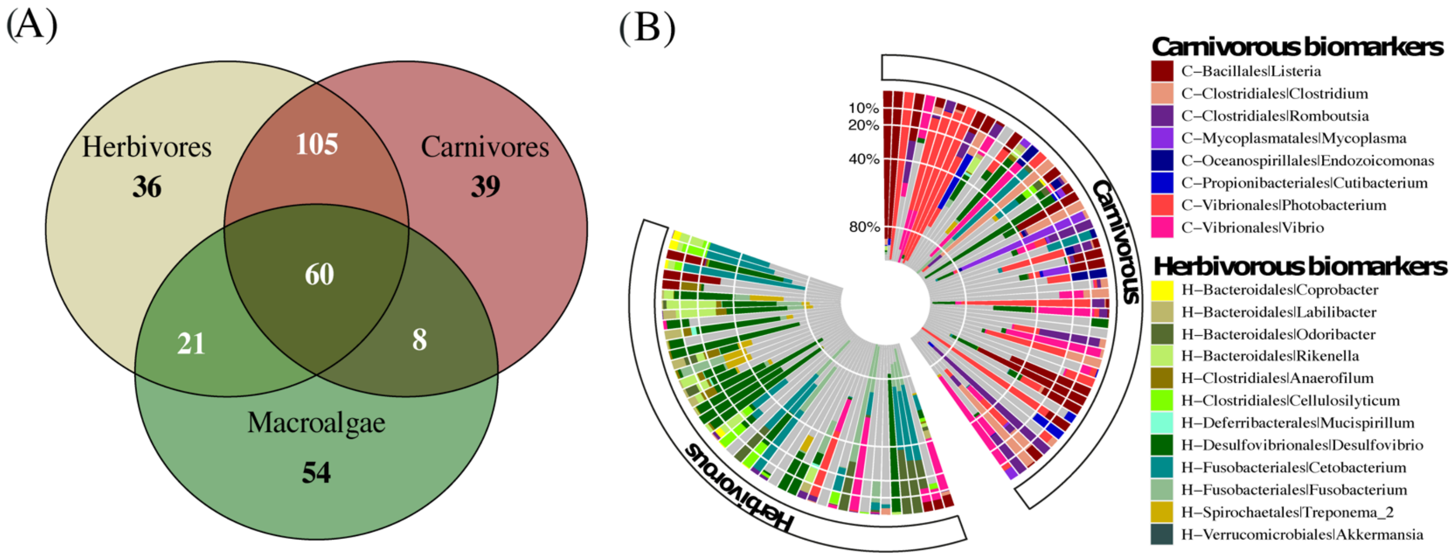

Figure S6: Venn diagram of the core ASVs between herbivores, carnivores and macroalgae bacteriomes (**A**) and Polar Histogram representing the relative abundance of the bacterial biomarkers for herbivores and carnivores bacteriomes (**B**). Biomarkers were delineated using a Linear discriminant analysis Effect Size (LEfSe) at Genus level. Bacterial taxa were considered as biomarkers for LDA score ⩾ 3. Contribution of the non-biomarkers phyla is represented in gray.

Supplementary Material

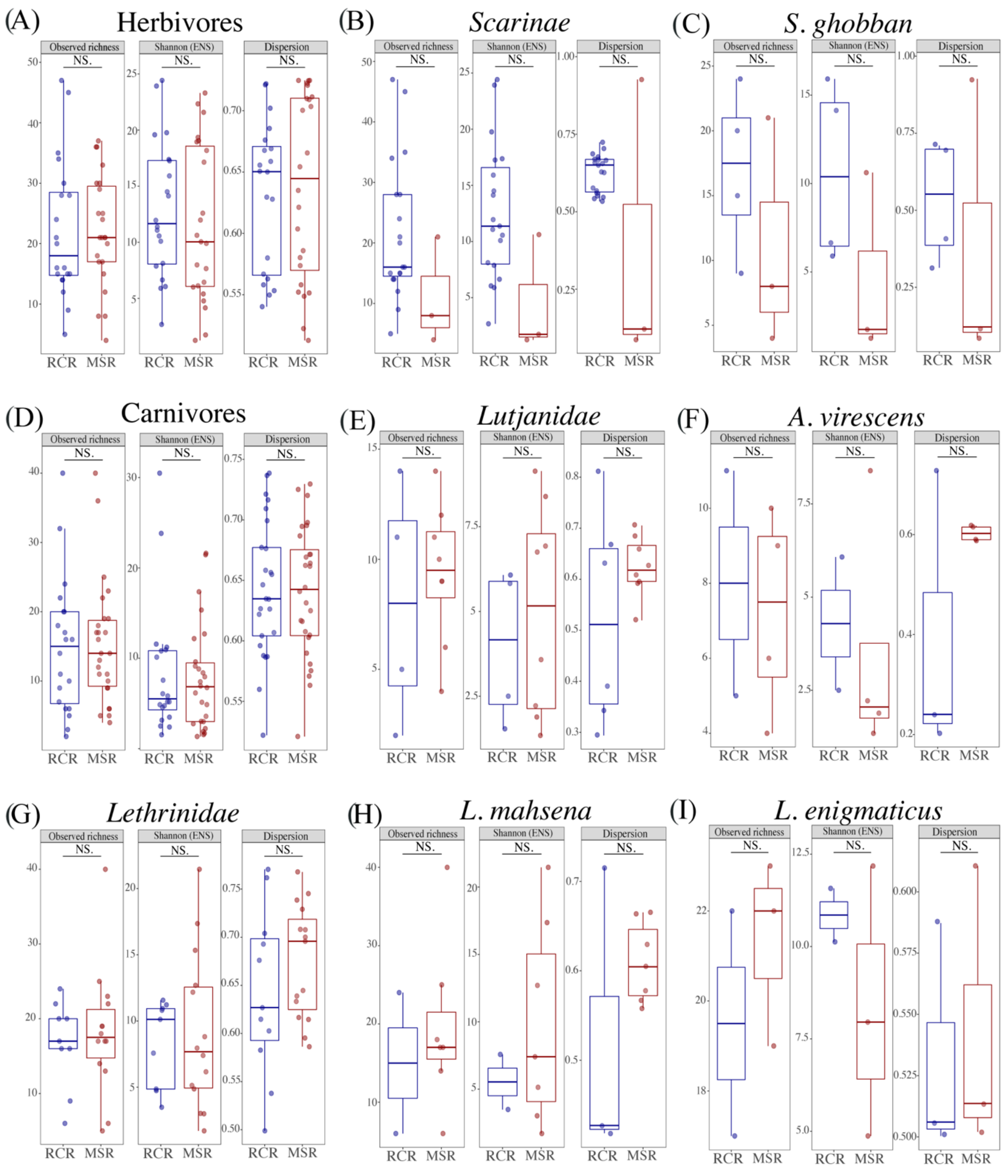

Figure S7: Boxplots representing the Alpha diversity, expressed as the observed richness and the Shannon's index H – exponentially transformed in effective number of species (ENS), and the dispersion (distance to centroïd for each sample type grouping) calculated for each bacteriome sample. Horizontal brackets indicate pairs which differ significantly: *** ⩽ 0.001 ; ** ⩽ 0.01 ; * ⩽ 0.05) or not (NS.) with a Wilcoxon test.

Supplementary Material

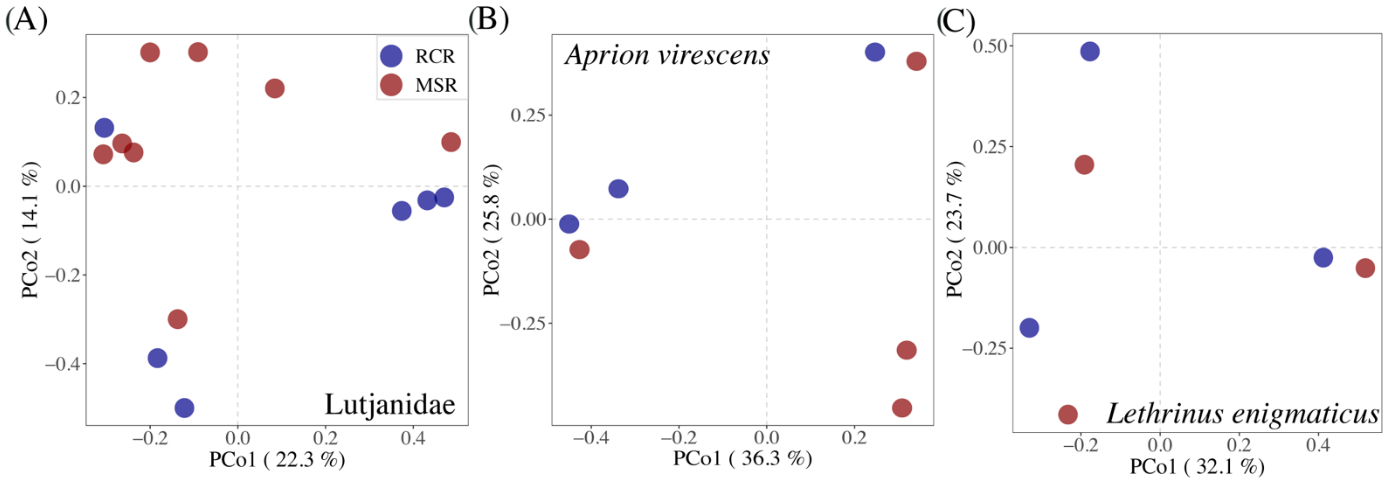

Figure S8: Principal coordinates analysis (PCoA) plot illustrating Bray-Curtis distances between pairs of bacteriome samples from the (**A**) *Lutjanidae*, (**B**) *A. virescens* and (**C**) *L. enigmaticus*. Bacteriomes are colored according to their origin: fish gut in orange and macroalgae in green. The p and R2 values of a PERMANOVA testing whether the origin of the samples have an influence are reported.
